# Scopoletin is a phytoalexin against *Alternaria alternata* in wild tobacco dependent on jasmonate signalling

**DOI:** 10.1093/jxb/eru203

**Published:** 2014-05-12

**Authors:** Huanhuan Sun, Lei Wang, Baoqin Zhang, Junhong Ma, Christian Hettenhausen, Guoyan Cao, Guiling Sun, Jianqiang Wu, Jinsong Wu

**Affiliations:** ^1^Key Laboratory of Economic Plants and Biotechnology, Kunming Institute of Botany, Chinese Academy of Sciences, Lanhei Road 132, 650201, Kunming, China; ^2^Dalian Institute of Chemical Physics, Chinese Academy of Sciences, 457 Zhongshan Road, Dalian 116023, China; ^3^Yunnan Academy of Tobacco Agricultural Science, Yuantong Street 33, 650031, Kunming, China

**Keywords:** *Alternaria alternata*, *feruloyl-CoA 6ʹ -hydroxylase* 1 (*F6ʹH1*), jasmonic acid (JA), MYC2, *Nicotiana attenuata*, scopoletin, virus-induced gene silencing (VIGS).

## Abstract

*Alternaria alternata* (tobacco pathotype) is a necrotrophic fungus causing severe losses in *Nicotiana* species by infection of mature leaves. Similar to what has been observed in cultivated tobacco, *N. tabacum*, young leaves of wild tobacco, *N. attenuata*, were more resistant to *A. alternata* than mature leaves, and this was correlated with stronger blue fluorescence induced after infection. However, the nature of the fluorescence-emitting compound, its role in defence, and its regulation were not clear. Silencing *feruloyl-CoA 6ʹ-hydroxylase 1* (*F6ʹH1*), the gene encoding the key enzyme for scopoletin biosynthesis, by virus-induced gene silencing (VIGS) revealed that the blue fluorescence was mainly emitted by scopoletin and its β-glycoside form, scopolin. Further analysis showed that scopoletin exhibited strong antifungal activity against *A. alternata in vitro* and *in vivo*. Importantly, jasmonic acid (JA) levels were highly elicited in young leaves but much less in mature leaves after infection; and fungus-elicited scopoletin was absent in JA-deficient plants, but was largely restored with methyl jasmonate treatments. Consistent with this, plants strongly impaired in JA biosynthesis and perception were highly susceptible to *A. alternata* in the same way scopoletin/scopolin-depleted VIGS F6ʹH1 plants. Furthermore, silencing *MYC2*, a master regulator of most JA responses, reduced *A. alternata*-induced *NaF6ʹH1* transcripts and scopoletin. Thus, it is concluded that JA signalling is activated in *N. attenuata* leaves after infection, which subsequently regulates scopoletin biosynthesis for the defence against *A. alternata* partly through MYC2, and higher levels of scopoletin accumulated in young leaves account for their strong resistance.

## Summary

This study demonstrates that wild tobacco *Nicotiana attenuata* plants accumulate the phytoalexin scopoletin to defend against the necrotrophic fungus *Alternaria alternata* in a JA signalling-dependent manner.

## Introduction

In many plant–pathogen systems, the resistance of plants usually depends on the developmental stage at which the plant is infected. Plants are generally more susceptible to disease in early than in late phases, including rice against *Xanthomonas oryzae*, tobacco to *Phytophthora parasitica*, and *Arabidopsis* to *Pseudomonas syringae* (Century *et al.*, 1999; Hugot *et al.*, 1999; Kus *et al.*, 2002; Develey-Riviere and Galiana, 2007). However, this is not true in the *Nicotiana tabacum*–*Alternaria alternata* interaction (Cheng and Sun, 2001; Zhang *et al.*, 1998).

*Alternaria alternata* (tobacco pathotype) is a necrotrophic fungus causing brown spot disease in *N. tabacum* (LaMondia, 2001). The disease usually occurs in mature leaves (Zhang *et al.*, 1998; Cheng and Sun, 2001). It has been shown that young leaves of tobacco are highly resistant to *A. alternata*, while they gradually lose their resistance as leaves become mature (Zhang *et al.*, 1998; Cheng and Sun, 2001). However, the reason for this age-dependent susceptibility is not clear. It may involve regulation of *R* genes, activation of different phytohormone signalling pathways, and accumulation of antifungal chemicals (Develey-Riviere and Galiana, 2007).

By using wild tobacco, *Nicotiana attenuata*, as a model plant, it was also observed that young source–sink transition leaves are more resistant to *A. alternata* than all fully expanded leaves, which is correlated with less abscisic acid (ABA) accumulated in older leaves (Sun *et al.*, 2014). This ABA distribution pattern at least partially contributed to the observed age-dependent susceptibility, since ABA-dependent stomatal closure is required for the resistance to *A. alternata* in wild tobacco (Sun *et al.*, 2014).

Interestingly, strong blue fluorescence was observed around the infection zone of *N. attenuata* leaves under UV light in this study, suggesting that some specific secondary metabolites were accumulated after infection. Whether this *A. alternata*-induced blue fluorescence is due to scopoletin, a blue autofluorescent compound accumulating in *Nicotiana* species after pathogen attack (Chong *et al.*, 2002; El Oirdi *et al.*, 2010), is not known. More importantly, the intensity of the blue fluorescence was 2- to 3-fold higher in source–sink transition leaves than in fully expanded leaves, indicating that this accumulation pattern may account for the age-dependent susceptibility. However, the nature of this fluorescence-emitting compound, its role in defence, and its regulation were not known.

Phytoalexins are antimicrobial substances of low molecular weight produced by plants in response to pathogen attack, including camalexin, the major substance in *Arabidopsis*, kauralexin and zealexin in maize, and scopoletin and capsidiol in *N. tabacum* (Ahuja *et al.*, 2012).

Scopoletin, a phenolic coumarin deriving from the phenylpropanoid pathway with strong blue fluorescence under UV light (Kai *et al.*, 2006, 2008), can be isolated from many plant species (Murray *et al.*, 1982), and was proposed as an important phytoalexin against microbial pathogens (Gnonlonfin *et al.*, 2012). Using T-DNA insertion mutants of *caffeoyl CoA O-methyltransferase1* and *feruloyl-CoA 6ʹ-hydroxylase 1* (*F6ʹH1*) in *Arabidopsis*, Kai *et al.* (2008) demonstrated that scopoletin biosynthesis is strongly dependent on F6ʹH1, and feruloyl-CoA is the key precursor. In addition, scopoletin levels are also influenced by the levels of scopoletin glucosyltransferase in tobacco (Chong *et al.*, 2002; Gachon *et al.*, 2004; Simon *et al.*, 2014).

In good agreement with its role in defence, scopoletin increases its level dramatically after fungal challenge, and exhibits fungitoxicity *in vitro* (Goy *et al.*, 1993; Garcia *et al.*, 1995; Valle *et al.*, 1997; Churngchow and Rattarasarn, 2001; Silva *et al.*, 2002; Carpinella *et al.*, 2005; El Oirdi *et al.*, 2010; Gnonlonfin *et al.*, 2012). Moreover, the resistance of some plant species against fungal pathogens is correlated with the rapidity and intensity of scopoletin accumulation (Goy *et al.*, 1993; Garcia *et al.*, 1995; Churngchow and Rattarasarn, 2001; El Oirdi *et al.*, 2010). These results represent a very important step in evaluating the defensive function of scopoletin; however, important experiments using scopoletin-depleted plants are still lacking. Ideally, the benefits of a putative defence trait should be determined in plants differing only in a single gene that controls the resistance trait and are otherwise identical (Bergelson *et al.*, 1996). In this study, virus-induced gene silencing (VIGS) of *F6ʹH1* was used to manipulate the production of scopoletin in order to investigate its role in the resistance of *N. attenuata* to *A. alternata*.

Here it is reported that *A. alternata*-induced blue fluorescence in *N. attenuata* leaves was mainly due to scopoletin and scopolin. Scopoletin possessed antifungal activity against the fungus *in vitro* and *in vivo*, and its production was demonstrated to be dependent on jasmonic acid (JA) signalling. Higher levels of this JA-dependent scopoletin in young leaves were one of the main reasons for their strong resistance.

## Materials and methods

### Plant and fungal materials

Seeds of the 31st generation of an inbred line of *N. attenuata* were used as the wild-type (WT) genotype. Stably transformed lines of ir*NaAOC* (Kallenbach *et al.*, 2012), ir*NaCOI1* (Paschold *et al.*, 2007), and ir*NaMPK4* (Hettenhausen *et al.*, 2012) were used as plants that were silenced in the expression of *N. attenuata allene oxide cyclase* (Na*AOC*; the gene encoding the key enzyme of JA biosynthesis), *NaCOI1* (encoding the JA-Ile receptor), and *NaMPK4*. Seed germination and plant growth were conducted as described by Krügel *et al*. (2002).

*Alternaria alternata* were grown and inoculated as described by Sun *et al.* (2014).

### Isolation of *NaF6ʹH1*

The 1275bp cDNA sequence of *NaF6ʹH1* (accession no. KF771989) was amplified by primers Js144_F, 5ʹ-ACAAAAATG CCTACTACAGTCTCA-3ʹ; and Js145_R, 5ʹ- TCCAGTCCAAAAT TCAGACACCT-3ʹ, the design of which was based on the sequence similarity to the tobacco expressed sequence tag (EST) EB446976, the EST FS420915, and *AtF6ʹH1* (At3g13610). Subsequently the cDNA fragment was cloned into a pMD18-T vector (Takara, www.takara.com.cn) and sequenced.

### Generation of VIGS plants

A 235bp fragment of the *NaF6ʹH1* cDNA sequence, amplified by primers JS138_F, 5ʹ- CAAAGAGGATTAACGTTAACTACTA-3ʹ; and JS140_R, 5ʹ- CTCTTGTATCGTCCATTGCTCATTAT-3ʹ was cloned into pTV00 (Ratcliff *et al.*, 2001), and *Agrobacterium tumefaciens* (strain GV3101) carrying these constructs were inoculated into *N. attenuata*, generating *NaF6ʹH1*-silenced plants (VIGS NaF6ʹH1).The *A. tumefaciens*-mediated transformation procedure was performed as described previously (Saedler and Baldwin, 2004; Wu *et al.*, 2008). To monitor the progress of VIGS, *phytoene desaturase* (PDS) was silenced; this is a gene that oxidizes and cyclizes phytoene to α- and β-carotene, compounds that are converted into the xanthophylls of the antenna pigments of the photosystems of plants. Silencing PDS eventually results in the visible bleaching of green tissues (Saedler and Baldwin, 2004; Wu *et al.*, 2008) ~2–3 weeks after the inoculation. When the leaves of *PDS*-silenced plants began to bleach and plants started bolting, the youngest rosette leaves of VIGS NaF6ʹH1 and empty vector-inoculated plants (EV plants) were selected for further experiments as source–sink transition leaves were hard to distinguish in VIGS plants. Around 40 plants were inoculated with EV and VIGS NaF6ʹH1 constructs, five biological replicates per genotype were used for experiments, and all VIGS experiments were repeated twice.

VIGS NaMYC2 plants were generated as described by Woldemariam *et al.* (2013)

### Bioassays for the inhibition of *A. alternata* growth by scopoletin *in vitro* and *in vivo*

The inhibition of *A. alternata* mycelium growth by scopoletin *in vitro* was tested in Petri dishes by subculturing a mycelium plug of 3mm diameter on PDA (potato dextrose agar) medium containing various concentrations of scopoletin for 6 d in the dark at 25 °C. A 50mg aliquot of scopoletin (Sigma) was dissolved in 5ml of methanol, and then added to the PDA medium at concentrations of 0, 48, 96, 240, and 480 μg ml^–1^. PDA plates supplied with 1% methanol served as controls. The area of mycelium growth was recorded every 2 d.

To test the role of scopoletin *in planta*, fully expanded +3 leaves (Sun *et al.*, 2014) were supplied through petiole feeding for 7h with 0, 100, and 500 μM scopoletin and then used for infection. These leaves usually had less fungus-induced blue fluorescence and were more susceptible to *A. alternata.* As indicators of the severity of disease symptoms, the diameters of the developing lesions at 5 days post-infection (dpi) were recorded.

### Quantification of blue fluorescence intensity, scopoletin, and scopolin

Leaf samples of ~0.1g were harvested from the inoculation site, and were ground in liquid nitrogen. A 1ml aliquot of 70% methanol with 1000ng ml^–1^ internal standard 4-methylumbelliferone was added to each sample. Supernatants were collected after vortexing and centrifugation at 15 000 *g* for 20min. Samples were then subjected to analysis by microplate reader and high-performance liquid chromatography–tandem mass spectrometry (HPLC-MS/MS; Thermo Scientific TSQ Quantum Access MAX).

The blue fluorescence intensities were determined by a microplate reader (Tecan infinite M200 PRO) at an excitation wavelength of 320nm and an emission wavelength of 420nm by comparing them with a standard curve of scopoletin (Sigma)

The levels of scopoletin and scopolin were determined by HPLC-MS/MS according to a method modified from Kai *et al.* (2006). Samples were separated by a Hyperil gold C18 column (Thermo), with H_2_O containing 0.1% (v/v) formic acid as solvent A and methanol containing 0.1% (v/v) formic acid as solvent B, at a ﬂow rate of 0.2ml min^–1^. Elution was started with isocratic conditions of 15% solvent B for 2min, followed by a linear gradient ﬂow up to 55% within 18min. The detection of scopoletin was set at *m/z* 193/133, scopolin at *m/z* 355/193, and 4-methylumbelliferone at *m/z* 177/77. The levels of scopoletin and scopolin were quantified by comparing their peak area with those of the internal standard.

### Analysis of JA

JA was extracted and quantified by LC-MS/MS as described by Wu *et al.* (2008).

### Real-time PCR

Total RNA was extracted from ground leaf samples using TRIzol reagent (Invitrogen) following the manufacturer’s instructions. For quantitative PCR analysis, five replicate biological samples were used. cDNA was synthesized from 500ng of total RNA with reverse transcriptases (Thermo Scientific, http://www.thermoscientificbio.com). Real-time PCR was performed as described (Sun *et al.*, 2014) on a Biorad CFX Connect qPCR System (Biorad, http://www.bio-rad.com) with iTaq Universal SYBR Green Supermix (Biorad) and gene-specific primers (Supplementary Table S1 available at *JXB* online) according to the manufacturer’s instructions.

## Results

### *Alternaria alternata*-induced blue fluorescence and *NaF6ʹH1* transcripts in *N. attenuata*

Previously, it was reported that young source–sink transition leaves (0 leaves) of wild tobacco *N. attenuata* are more resistant to *A. alternata* than all the fully expanded leaves including +3 leaves (Sun *et al.*, 2014). In addition to ABA signalling-mediated stomatal immunity (Sun *et al.*, 2014), it was hypothesized that young source–sink transition leaves may accumulate higher amounts of antifungal chemicals than fully expanded leaves do after infection, and this accumulation pattern may account for the age-dependent susceptibility.

**Fig. 1.**
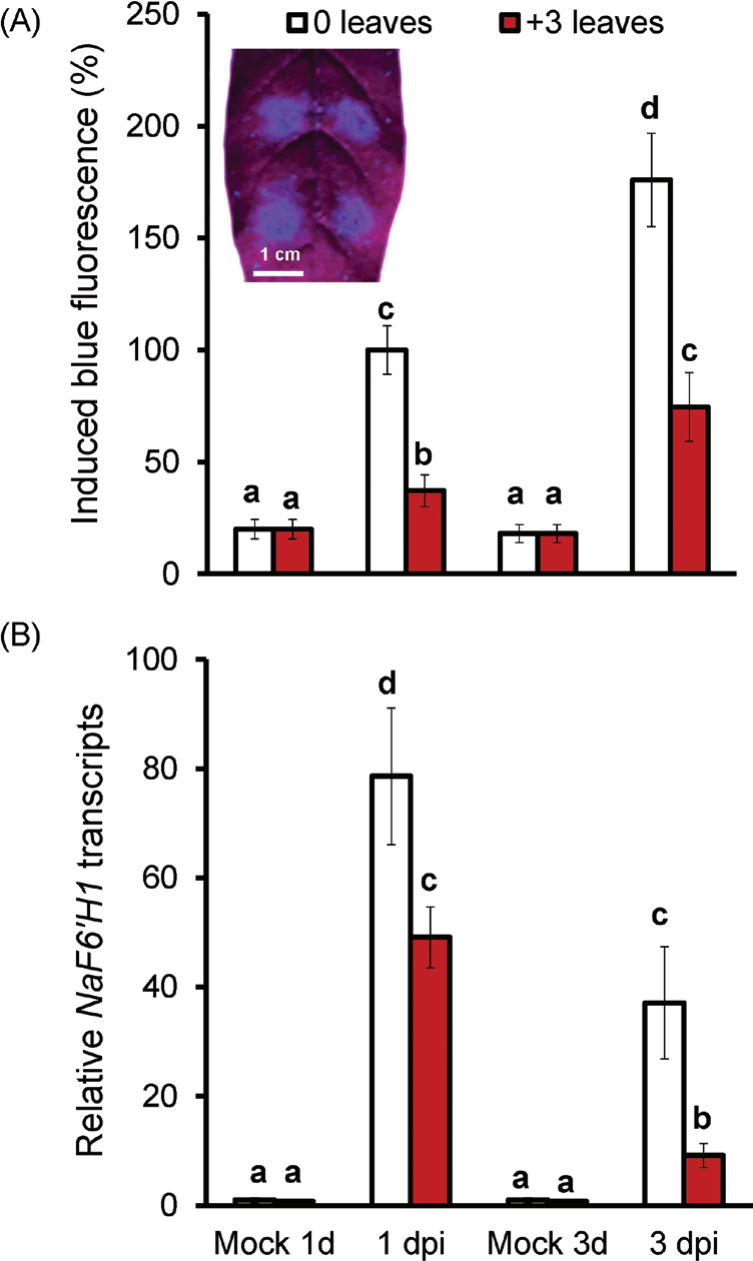
*Alternaria alternata*-induced blue fluorescence and *NaF6′H1* transcripts in *N. attenuata*. (A) Relative blue fluorescence intensity induced by *A. alternata* in four replicates of 0 and +3 leaves at 1 and 3 dpi. The mean (±SE) level of the intensity in 0 leaves at 1 dpi was arbitrarily set as 100%. Inset: one 0 leaf was inoculated with four agar plugs with active *A. alternata* mycelium for 1 d, and then agar plugs were removed in order to photograph the leaves under UV light. Strong blue fluorescence was observed around the four inoculation sites. (B) Mean (±SE) *NaF6ʹH1* transcripts were measured by real-time PCR in five replicates of 0 and +3 leaves infected with *A. alternata* at 1 and 3 dpi. The level of *NaF6ʹH1* transcripts in 0 leaves with mock 1 d treatment was arbitrarily set as 1. Leaves inoculated with an PDA agar plug only (without *A. alternata*) for 1 and 3 d served as controls. Different letters indicated significant differences between each treatment group (one-way ANOVA, *P*<0.05).

Strong blue fluorescence was observed around the infection zones in 0 leaves at 1 dpi, and the fluorescence intensity was even higher at 3 dpi (Fig. 1A). However, in fully expanded +3 leaves, which are more susceptible to *A. alternata* (Sun *et al.*, 2014), only around one-third of the blue fluorescence intensity that accumulated in 0 leaves was observed at both 1 and 3 dpi (Fig. 1A), indicating that some compounds capable of emitting blue fluorescence under UV light were highly induced after fungal challenge, and these chemicals may contribute to the resistance to *A. alternata*.

Kai *et al.* (2008) demonstrated that scopoletin biosynthesis is strongly dependent on *F6ʹH1* in *Arabidopsis* roots. A cDNA which displayed a high sequence similarity to *F6ʹH1* in *Arabidopsis* was cloned in *N. attenuata*, and it is referred to as *NaF6ʹH1* (GenBank accession no. KF771989; Supplementary Fig. S1 at *JXB* online). *NaF6ʹH1* transcripts were dramatically induced after infection (Fig. 1B); young 0 leaves at 1 dpi accumulated ~80 times more *NaF6ʹH1* transcripts than did mock controls. Transcripts of *NaF6ʹH1* were also highly induced in +3 leaves, but its levels were significantly lower than those of 0 leaves at both 1 and 3 dpi.

### Silencing *NaF6ʹH1* reduces scopoletin levels and plant resistance

**Fig. 2.**
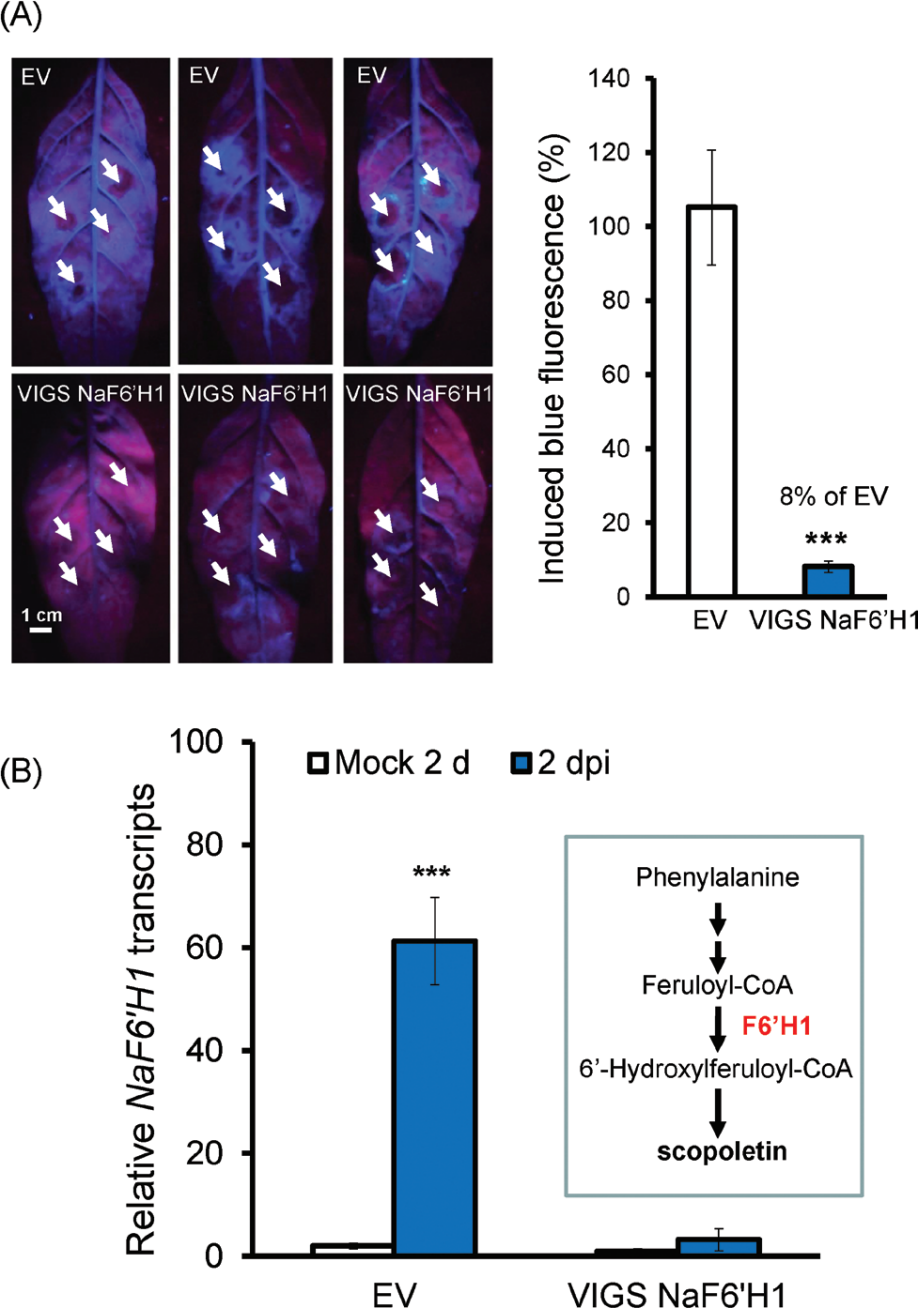
*Alternaria alternata*-elicited blue fluorescence and *NaF6ʹH1* transcripts in EV and VIGS NaF6′H1 plants. (A) Left: three independent young leaves from EV and VIGS NaF6ʹH1 plants were infected with *A. alternata*. Photographs were taken from their underside under UV light at 5 dpi; strong blue fluorescence was emitted around the four inoculation sites (indicated by white arrows) of each leaf in EV but not in VIGS NaF6ʹH1 plants. Right: quantification of blue fluorescence intensity induced by *A. alternata* at 5 dpi in four replicates of leaves of EV and VIGS NaF6ʹH1 plants. The level of the intensity in EV plants was arbitrarily set as 100%. Asterisks indicate the level of significant differences between EV and VIGS NaF6ʹH1 plants (Student’s *t*-test: ***P<0.005). (B) Mean (±SE) relative *A. alternata*-induced *NaF6ʹH1* transcript levels as measured by real-time PCR in four replicates of leaves of EV and VIGS NaF6′H1 plants at 2 dpi. Inset: schematic depiction of the scopoletin biosynthetic pathway. Asterisks indicate the level of significant differences between mock and infected samples in EV plants (Student’s *t*-test: ***P<0.0001).

To understand whether the *A. alternata*-induced blue fluorescence was emitted by scopoletin, *NaF6ʹH1*, the candidate key enzyme gene for scopoletin biosynthesis, was silenced (Fig. 2B) to generate scopoletin-depleted *N. attenuata* plants by VIGS.

At 5 dpi, strong blue fluorescence was observed around the infection zone of young leaves in plants transformed with the empty vector (EV plants), but only 8% of the fluorescence intensity of EV plants was detected in plants transformed with the *NaF6ʹH1*-silenced construct (VIGS NaF6ʹH1 plants; Fig. 2A). The transcriptional levels of *NaF6ʹH1* in VIGS NaF6ʹH1 plants did not increase significantly after elicitation, and were reduced by 95% when compared with EV plants at 2 dpi (Fig. 2B), indicating that silencing of *NaF6ʹH1* was effective and the fungus-elicited fluorescence was dependent on NaF6ʹH1.

**Fig. 3.**
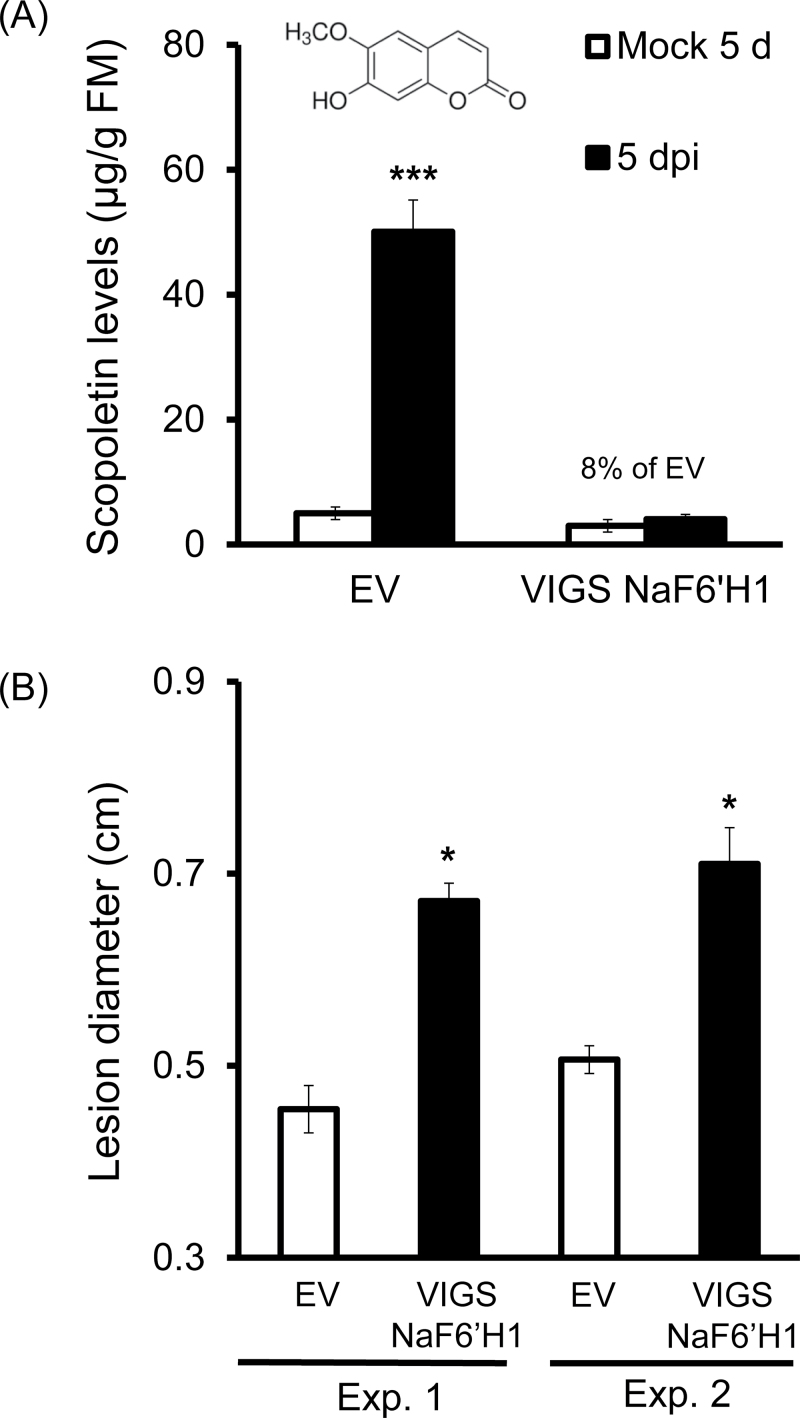
Silencing *NaF6ʹH1* reduced *A. alternata*-induced scopoletin levels and plant resistance. (A) Mean (±SE) scopoletin levels were determined by LC-MS/MS in five replicates of young leaves of EV and VIGS NaF6ʹH1 infected with *A. alternata* for 5 d. Asterisks indicate the level of significant differences between mock and infected samples in EV plants (Student’s *t*-test: ***P<0.005). (B) Mean (±SE) diameter of necrotic lesions in four replicates of young leaves of EV and VIGS NaF6ʹH1 infected with *A. alternata* for 7 d. Two independent VIGS experiments are presented showing similar results. The asterisk indicates the level of significant difference between EV and VIGS NaF6ʹH1 leaves (Student’s *t*-test: *P<0.05).

The amounts of fungus-elicited scopoletin and its β-glycoside scopolin, both of which are capable of emitting blue fluorescence, were quantified by LC-MS/MS. About 50 μg of scopoletin g^–1^ fresh leaf mass were detected in EV leaves at 5 dpi; in contrast, only ~4 μg g^–1^ fresh leaf mass were found in VIGS NaF6ʹH1 leaves with the same treatment (Fig. 3A). At the same time, scopolin accumulated to 72.5 μg g^–1^ fresh leaf mass in EV leaves but only one-fifth of these levels were detected in VIGS NaF6ʹH1 leaves (Supplementary Fig. S2A at *JXB* online). The total blue fluorescence intensity was also quantified by a microreader, and the results revealed that the total fluorescence intensity was equivalent to the amount of chemicals emitted by ~110 μg g^–1^ fresh leaves of scopoletin, suggesting that the *A. alternata*-induced blue fluorescence was mainly due to scopoletin and scopolin.

Importantly, two independent VIGS experiments showed that pathogen-induced necrotic lesions were significantly larger in VIGS NaF6ʹH1 plants than in EV plants when the youngest rosette leaves were infected with *A. alternata* (Fig. 3B).

All these results strongly indicated that the blue fluorescence induced by *A. alternata* was NaF6ʹH1 dependent, and was mainly due to scopoletin and scopolin, and that these compounds are important for the resistance against *A. alternata*.

### The inhibition of *A. alternata* growth *in vitro* and *in vivo* by scopoletin

**Fig. 4.**
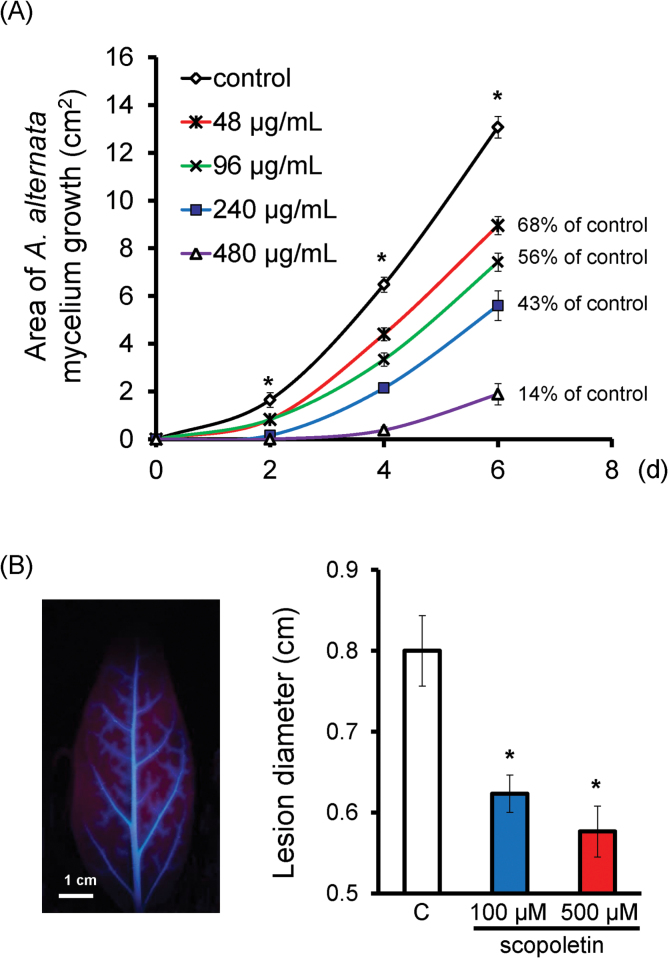
Inhibition of *A. alternata* growth *in vitro* and *in vivo*. (A) Area of *A. alternata* mycelium growth on PDA supplied with 0, 48, 96, 240, or 480 μg ml^–1^ scopoletin; data were collected every 2 d. (B) Left panel: a detached +3 leaf was fed with 500 μM scopoletin for 7h, and then the photograph was taken under UV light. Right panel: mean diameter (±SE) of necrotic lesions in four replicates of +3 leaves at 5 dpi, which were pre-treated with water or with 100 μM or 500 μM scopoletin for 7h before inoculation. Asterisks indicate the level of significant difference between control and treated samples (Student’s *t*-test: *P<0.05).

To confirm further the role of scopoletin in the resistance of *N. attenuata* to *A. alternata*, it was next tested whether it could restrict fungal growth on PDA medium and *in planta*. Mycelium growth of *A. alternata* on PDA plates with 48 μg ml^–1^ or 96 μg ml^–1^ scopoletin, concentrations more or less equal to physiological levels, was decreased to 68% or 56% of that of control plates, and it was reduced further to 43% and 14% in plates supplied with 240 μg ml^–1^ and 480 μg ml^–1^ scopoletin, respectively (Fig. 4A), confirming that scopoletin has a large impact on fungal growth *in vitro*, as reported (Garcia *et al.*, 1995; Valle *et al.*, 1997; Churngchow and Rattarasarn, 2001; Silva *et al.*, 2002; Carpinella *et al.*, 2005). When +3 leaves, which accumulate less scopoletin (Fig. 1A) and are more susceptible to *A. alternata* (Sun *et al.*, 2014), were fed with 100 μM or 500 μM scopoletin through the petioles for 7h and then used for infection, they developed significantly smaller lesions than control leaves (Fig. 4B), indicating that exogenously supplied scopoletin enhanced plant resistance to *A. alternata*.

### *Alternaria alternata*-induced scopoletin biosynthesis is strongly dependent on JA but not ABA signalling

Previously it was shown that ABA signalling plays an important role in the resistance of *N. attenuata* to *A. alternata* through stomatal immunity (Sun *et al.*, 2014). Feeding with 10 μM ABA for 4h, which is effective in inducing stomatal closure responses and resistance to *A. alternata* (Sun *et al.*, 2014), had little effect on *A. alternata*-elicited scopoletin levels (Supplementary Fig. S3A at *JXB* online). The fungus-induced blue fluorescence intensity at 1 dpi was slightly reduced in 0 leaves but not in +3 leaves in irNaMPK4 (ABA-insensitive) plants (Supplementary Fig. S3B). These results suggested that ABA signalling had a minor effect on the *A. alternata*-elicited scopoletin production.

It is commonly believed that plant defence responses to necrotrophic pathogens are regulated by JA signalling (Glazebrook, 2005). Thus pathogen-induced JA levels were measured by LC-MS/MS, and JA mutants generated previously by RNA interference (RNAi) in *N. attenuata* were also selected to verify the involvement of JA signalling in scopoletin production and defence against *A. alternata*.

**Fig. 5.**
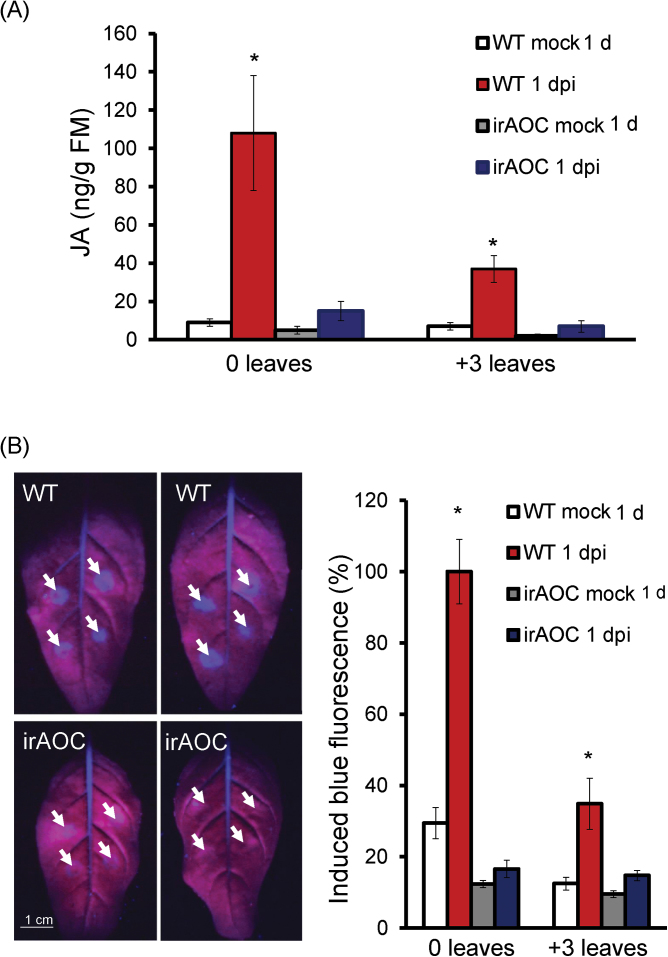
*Alternaria alternata*-induced JA is required for the fungus-elicited scopoletin production. (A) Mean (±SE) JA levels elicited by *A. alternata* at 1 dpi determined by LC-MS/MS in five replicates of 0 and +3 leaves of WT and irAOC plants. (B) Left: two 0 leaves from WT and irAOC plants were infected with *A. alternata* for 1 d. Photographs were taken from their underside under UV light; strong blue fluorescence was emitted around four inoculation sites (indicated by white arrows) of each leaf in WT plants but not in irAOC plants. Right: mean (±SE) relative blue fluorescence intensity induced by *A. alternata* at 1 dpi in five replicates of 0 leaves of WT or irAOC plants. The level of intensity in 0 leaves of WT plants was arbitrarily set as 100%. Asterisks indicate the level of significant differences between mock and infected samples (Student’s *t*-test: *P<0.05).

**Fig. 6.**
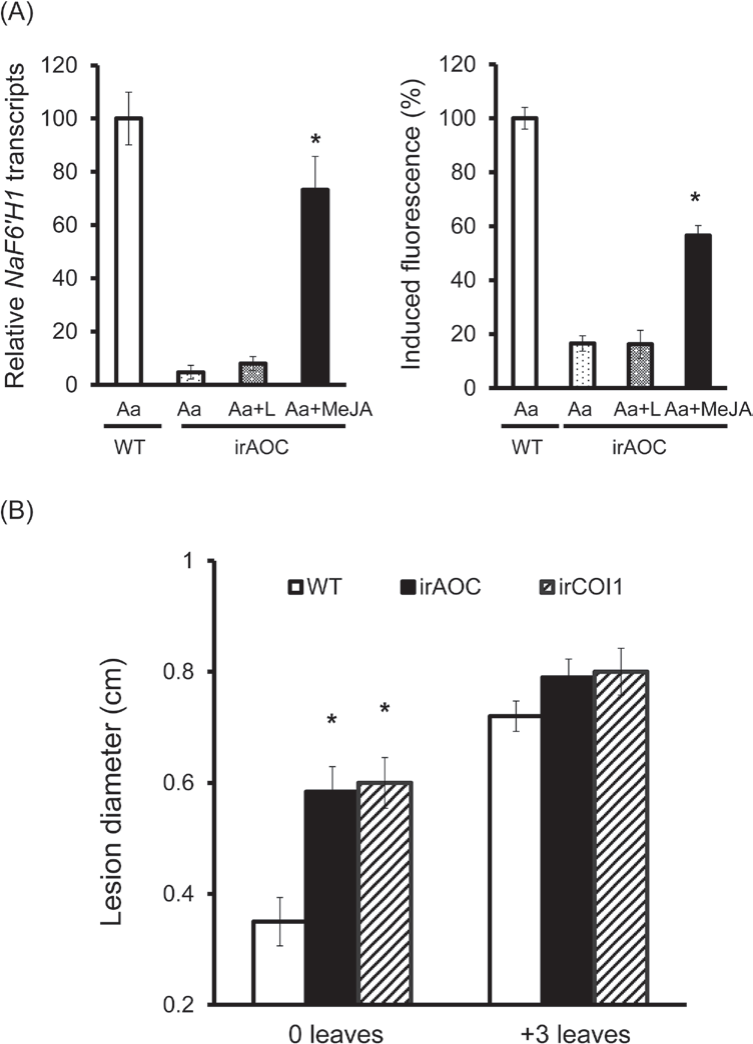
MeJA largely restores *A. alternata*-elicited responses in JA-deficient plants, and both JA biosynthesis and perception are required for plant resistance. (A) Left: mean (±SE) *NaF6ʹH1* transcripts as measured by real-time PCR in four replicates of 0 leaves of WT and irAOC plants infected with *A. alternata* for 1 d. Right: mean (±SE) relative blue fluorescence intensity induced by *A. alternata* at 1 dpi in five replicates of leaves of WT and irAOC plants. The levels of *NaF6′H1* transcripts and blue fluorescence intensity in 0 leaves of WT plants was arbitrarily set as 100%. All leaves were inoculated with *A. alternata* (Aa), five leaves of irAOC were additionally treated with lanolin (Aa+L), and another five leaves were treated with 150 μg of MeJA in lanolin paste (Aa+MeJA). Asterisks indicate the level of significant difference between lanolin and MeJA-treated leaves (Student’s *t*-test: *P<0.05). (B) Mean (±SE) diameter of necrotic lesions of five replicates of 0 and +3 leaves of WT, irCOI1, and irAOC plants at 5 dpi. Asterisks indicate the level of significant differences between WT and irCOI1 or irAOC plants (Student’s *t*-test: *P<0.05).

In response to *A. alternata* infection at 1 dpi, *N. attenuata* plants increased their JA levels dramatically in 0 leaves (increased from 10ng g^–1^ to 100ng g^–1^ fresh leaves; Fig. 5A), but much less in +3 leaves, where JA levels were also significantly elicited but were only around one-third of those of 0 leaves (Fig. 5A). However, the induction of JA was almost abolished in plants silenced in *AOC* (irAOC; Fig. 5A), which has been demonstrated to be the gene encoding the key enzyme leading to the formation of JA (Kallenbach *et al.*, 2012). Importantly, *A. alternata*-elicited blue fluorescence at 1 dpi was completely abolished in irAOC plants (Fig. 5B), indicating that the fungus-induced scopoletin production was dependent on JA. Indeed, not only the levels of blue fluorescence but also *NaF6ʹH1* transcripts were not elicited at all in irAOC plants (Fig. 6A). However, when irAOC plants were treated with methyl jasmonate (MeJA), which was rapidly metabolized to JA by methyl jasmonate esterase *in planta* (Wu *et al.*, 2008), the levels of both *A. alternata*-elicited blue fluorescence and *NaF6ʹH1* transcripts were largely restored (Fig. 6A). These results demonstrated that *A. alternata*-induced JA production was indeed very important for scopoletin biosynthesis.

To confirm further the role of JA signalling for fungus-induced scopoletin, plants silenced with COI1 previously generated by Paschold *et al.* (2007) were also tested. As expected, the fungus-elicited scopoletin levels in irCOI1 were the same as those in irAOC plants at 1 dpi, which were similar to the levels in WT mock controls (Supplementary Fig. S2B at *JXB* online), suggesting that the perception of JA signalling is also very important for the induction of scopoletin.

Consistent with the role of scopoletin in the resistance of *A. alternata*, irAOC and irCOI1 plants were also shown to be more susceptible to the fungus by developing larger lesions in 0 leaves (Fig. 6B), where more JA and scopoletin were elicited. In contrast, lesion diameters were almost similar in +3 leaves (Fig. 6B), most probably because JA signalling was less induced and overall scopoletin levels were lower in these leaves.

### MYC2 is involved in the regulation of scopoletin biosynthesis

**Fig. 7.**
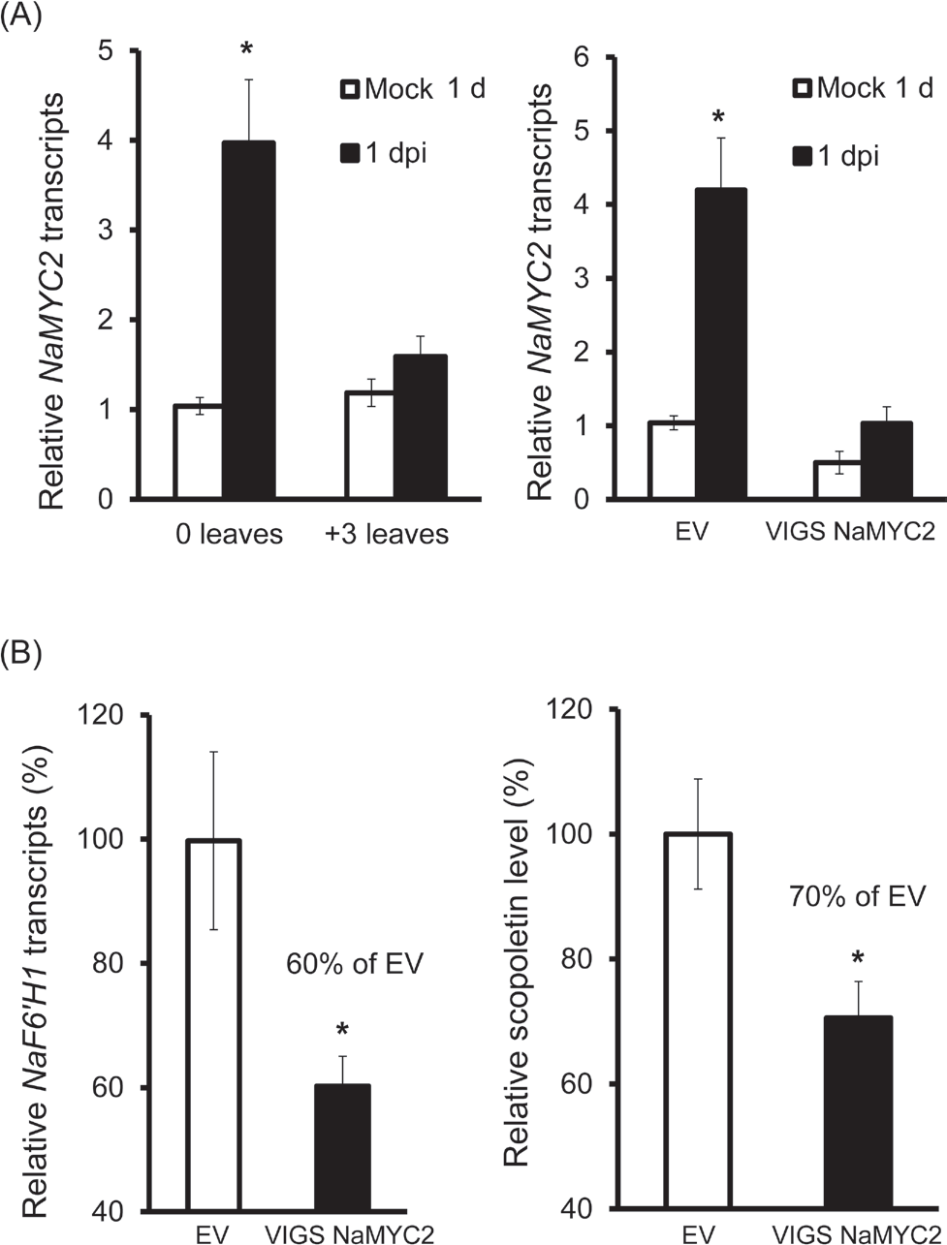
Silencing *NaMYC2* affects *A. alternata*-elicited levels of *NaF6ʹH1* transcripts and scopoletin. (A) Left: mean (±SE) *NaMYC2* transcripts were measured by real-time PCR in four replicates of 0 and +3 leaves of the WT at 1 dpi. Right: mean (±SE) *NaMYC2* transcripts were measured by real-time PCR in four replicates of the youngest rosette leaves of EV and VIGS *NaMYC2* plants at 1 dpi. (B) Left: mean (±SE) *NaF6ʹH1* transcripts were measured by real-time PCR in four replicates of the youngest rosette leaves of EV and VIGS *NaMYC2* plants at 1 dpi. Right: relative mean (±SE) scopoletin levels were determined by LC-MS/MS in five replicates of the youngest rosette leaves of EV and VIGS *NaMYC2* plants at 1 dpi. The level in EV plants was arbitrarily set as 100%. Asterisks indicate the level of significant differences between mock and infected plants at 1 dpi or between EV and VIGS Na*MYC2* plants (Student’s *t*-test: *P<0.05; ***P<0.0001).

MYC2, a member of the basic helix–loop–helix (bHLH) family of transcription factors, participates in the regulation of many JA-dependent defences against insect herbivores and pathogens (Lorenzo *et al.*, 2004; Dombrecht *et al.*, 2007; Woldemariam *et al.*, 2013). Consistent with the induction of JA at 1 dpi, the levels of MYC2 transcripts were increased to 4-fold in 0 leaves compared with mock control, but remained unchanged in +3 leaves (Fig. 7A, left panel).

To investigate whether *MYC2* was involved in the regulation of scopoletin biosynthesis, *MYC2* was also silenced by VIGS in this study. *Alternaria alternata* infection increased *MYC2* transcripts significantly by 4-fold as expected in EV plants, but not in VIGS *MYC2* plants at 1 dpi (Fig. 7A, right panel), suggesting that the *MYC2* gene was successfully silenced in VIGS *MYC2* plants. Meanwhile, *NaF6ʹH1* transcripts were reduced by 40%, and scopoletin levels were also reduced by 30% in VIGS *MYC2* plants when compared with EV plants at 1 dpi (Fig. 7B). These results indicated that MYC2 was involved in the regulation of scopoletin biosynthesis.

## Discussion

*Alternaria alternata* is a necrotrophic fungus causing brown spot disease in *N. tabacum* (LaMondia, 2001) and wild tobacco *N. attenuata* (Sun *et al.*, 2014). Similar to cultivated tobacco (Zhang *et al.*, 1998; Cheng and Sun, 2001), young leaves of *N. attenuata* are more resistant to *A. alternata* than mature leaves (Sun *et al.*, 2014). Previous work has demonstrated that ABA signalling-mediated stomata-based defence partially contributes to this age-dependent susceptibility (Sun *et al.*, 2014), and now the new data presented here reveal that a higher level of scopoletin accumulated in young leaves is another main reason for this.

Scopoletin, a phenolic coumarin, has been proposed to have an important role in defending against fungal pathogens (Goy *et al.*, 1993; Garcia *et al.*, 1995; Valle *et al.*, 1997; Churngchow and Rattarasarn, 2001; Silva *et al.*, 2002; Carpinella *et al.*, 2005; El Oirdi *et al.*, 2010; Gnonlonfin *et al.*, 2012). However, important evidence regarding whether scopoletin/scopolin-depleted plants were more susceptible was lacking. The present data strongly indicate that *N. attenuata* plants accumulate scopoletin and scopolin as defence compounds against *A. alternata*, since their levels were dramatically increased after *A. alternata* infection (Figs 1A, 3A); 0 leaves accumulated more and they were more resistant (Figs 1A, 6B); scopoletin exhibited fungal toxicity to *A. alternata in planta* and *in vitro* (Fig. 4); and, more importantly, scopoletin/scopolin-depleted plants, which were generated by silencing *NaF6ʹH1*, were more susceptible to *A. alternata* (Fig. 3B).

The results of silencing *NaF6ʹH1* also indicated that the *A. alternata*-elicited blue fluorescence was due mainly to scopoletin and scopolin. However, further analysis is needed to exclude the possibility of the involvement of other NaF6ʹH1-based courmarins including esculetin and esculin, which are also capable of emitting blue fluorescence under UV light. Because there is no direct evidence of an effect of scopolin on the restriction of fungal growth, whether scopolin plays a role in the resistance against *A. alternata* is still not known. However, it may be converted back to scopoletin *in planta*. In tobacco, silencing or overexpression of a scopoletin glucosyltransferase affects scopoletin and scopolin levels and also plant resistance to *Tobacco mosaic virus* (Chong *et al.*, 2002; Gachon *et al.*, 2004). It will be interesting to find the de-glucosylation enzyme responsible for the conversion of scopolin to scopoletin, and to test its role in defence.

The formation of scopoletin is induced by various stress or phytohormone treatments (Gnonlonfin *et al.*, 2012). Induction of cytokinin biosynthesis in tobacco enhances resistance to the hemibiotrophic pathogen *Pseudomonas syringae* pv *tabaci* by increasing scopoletin and capsidiol independent of JA and salicylic acid (SA) signalling (Grosskinsky *et al.*, 2011). However, whether cytokinin is also involved in the regulation of scopoletin in *N. attenuata* against *A. alternata* needs further investigation. In *Arabidopsis*, the levels of scopoletin in the shoots increase after treatment with 2,4-dichlorophenoxyacetic acid (2,4-D), whereas other plant hormones such as SA, MeJA, and kinetin have no effects (Kai *et al.*, 2006). Consistent with this, the present results also showed that MeJA alone cannot elicit scopoletin production (Supplementary Fig. S5 at *JXB* online), but these findings contradict what has been observed in tobacco suspension culture (Sharan *et al.*, 1998), suggesting that MeJA-induced responses in intact plants differ from those in cell cultures.

Compared with defence responses induced by SA, JA-regulated genes are more involved in the resistance against necrotrophic pathogens (Glazebrook, 2005); for example, JA-insensitive *coi1* mutants of *Arabidopsis* are highly susceptible to the necrotrophic fungi *Alternaria brassicicola* and *Botrytis cinerea* (Spoel *et al.*, 2007; Rowe *et al.*, 2010). To a large extent, *B. cinerea*-elicited camalexin is controlled by JA signalling (Rowe *et al.*, 2010). However, whether JA plays a role in the regulation of scopoletin in the resistance of *N. attenuata* to *A. alternata* is not known.

In wild tobacco *N. attenuata*, JA biosynthesis starts with the release of trienoic fatty acids [e.g. α-linolenic acid (18:3)] from membrane lipids in the chloroplast, which are oxidized by 13-lipoxygenases (NaLOX3) to form (13S)-hydroperoxy-18:3 (Halitschke and Baldwin, 2003). This molecule is the substrate for allene oxide synthase and subsequently AOC (Kallenbach *et al.*, 2012). 12-Oxophytodienoic acid (OPDA) is formed and transported into the peroxisome where it is oxidized, forming JA. In the cytosol, JA-Ile, the bioactive form of jasmonate, is produced by the conjugation of JA to isoleucine by JASMONATE RESISTANT (JAR) (Kang *et al.*, 2006; Wang *et al*., 2007, 2008). JA-Ile activates the SCF^C^°^I1^–JAZ complex (Chini *et al.*, 2007; Thines *et al.*, 2007), leading to the release of transcription factors such as MYC2, which is repressed by JAZ in the absence of JA (Lorenzo *et al.*, 2004; Dombrecht *et al.*, 2007; Woldemariam *et al.*, 2013).

The present data strongly support the idea that JA signalling plays an important role in *N. attenuata* in defending against the necrotrophic fungus *A. alternata* by regulating NaF6ʹH1-dependent scopoletin biosynthesis. JA production was increased after fungal challenge (Fig. 5A); 0 leaves, which were more resistant to *A. alternata*, accumulated more JA and more scopoletin (Figs 1A, 5A); and plants strongly impaired in JA biosynthesis (irAOC) and perception (irCOI1) could not respond to *A. alternata* infection by increasing scopoletin production (Fig. 5B; Supplementary Fig. S2B at *JXB* online), and these plants were highly susceptible to *A. alternata* (Fig. 6B). In addition, exogenous MeJA can partially restore the levels of *A. alternata*-induced *NaF6ʹH1* transcripts and scopoletin production in JA-deficient irAOC plants (Fig. 6A).

MYC2 is a member of bHLH family of transcription factors, and has emerged as a master regulator of most aspects of the JA signalling pathway in *Arabidopsis* and *N. attenuata* (Lorenzo *et al.*, 2004; Dombrecht *et al.*, 2007; Woldemariam *et al.*, 2013), by binding to the G-box motif and its variants found in MYC2 target promoters (Godoy *et al.*, 2011; Kazan and Manners, 2013). In *Arabidopsis*, two major branches of the JA signalling pathway are recognized: the MYC2 branch and the ERF (ethylene response factor) branch. In general, the ERF branch is associated with enhanced resistance to necrotrophic pathogens (Berrocal-Lobo *et al.*, 2002; Lorenzo *et al.*, 2003), whereas the MYC2 branch is associated with the wound response and defence against insect herbivores (Lorenzo *et al.*, 2004; Kazan and Manners, 2008;Verhage *et al.*, 2010). The MYC2-regulated branch antagonizes the ERF branch, and accordingly the *myc2* mutant increased resistance to *B. cinerea* (Lorenzo *et al.*, 2004). In *N. attenuata*, a T/G-box 5ʹ-AACGTG-3ʹ, one of the motifs recognized by MYC2 in the promoter region of many JA-responsive genes (Boter *et al.*, 2004; Godoy *et al.*, 2011), was also detected in the *NaF6ʹH1* promoter (Supplementary Fig. S4 at *JXB* online). Consequently, silencing *MYC2* in *N. attenuata* affected fungus-induced *NaF6ʹH1* transcripts and scopoletin production (Fig. 7), indicating that unlike in *Arabidopsis*, *NaMYC2* plays a role in the resistance of *N. attenuata* to the necrotrophic fungus *A. alternata* by regulating scopoletin biosynthesis. However, silencing *NaMYC2* did not abolish fungus-induced scopoletin, suggesting that some other unknown transcriptional factors dependent on JA are also involved.

Previous work has shown that ABA is involved in the resistance of *N. attenuata* to *A. alternata*, and, like JA, higher levels of ABA were also detected in young leaves (Sun *et al.*, 2014). Whether fungus-induced ABA cross-talks with JA needs further investigation. In *Arabidopsis*, ABA has been proposed to have an important role in the activation of defences against the oomycete *Pythium irregulare* by affecting JA biosynthesis (Adie *et al.*, 2007). It will be interesting to test whether *A. alternata*-elicited JA levels are influenced in ABA-deficient *N. attenuata* plants.

**Fig. 8.**
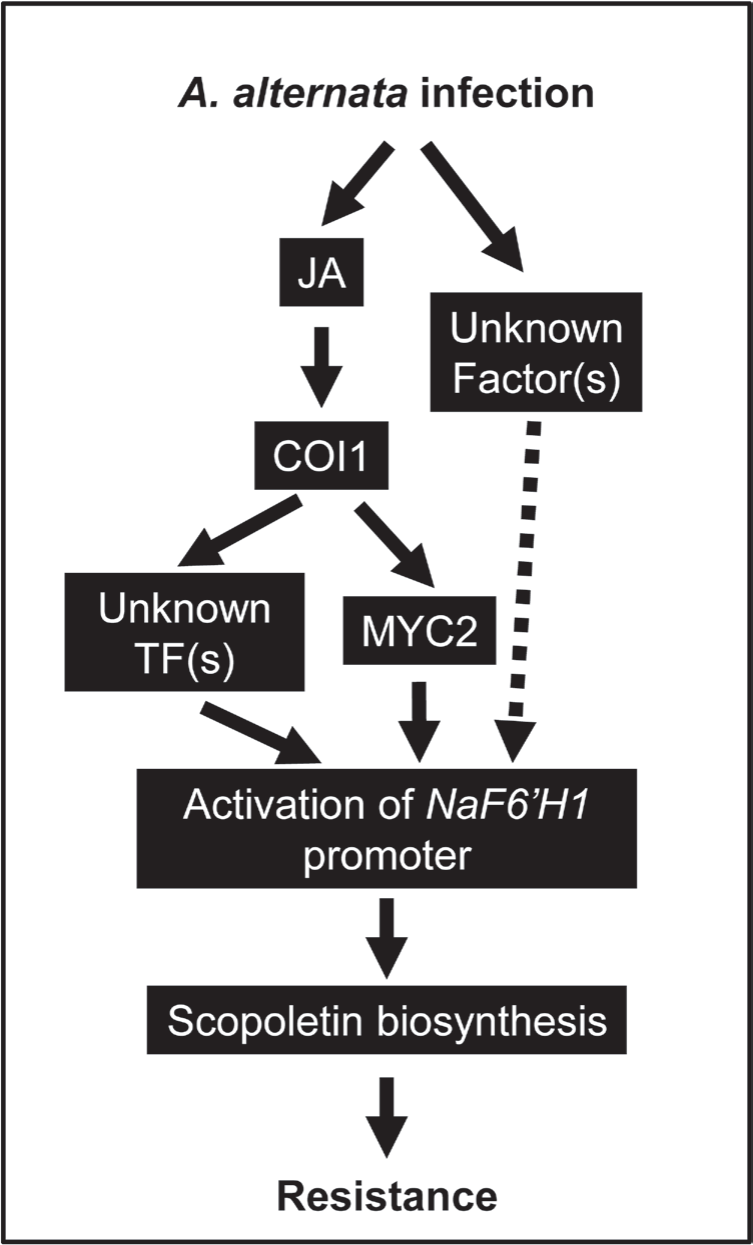
Working model of the regulation of *A. alternata*-induced scopoletin production by JA signalling. After *A. alternata* infection, the JA signalling pathway is activated and *NaF6ʹH1*, coding for the key enzyme in scopoletin biosynthesis, is trans-activated by the binding of MYC2 and some other unknown proteins to the T/G-box of the *NaF6ʹH1* promoter; the biosynthesis of scopoletin around the infection sites finally affects the resistance of *N. attenuata* to *A. alternata*. However, without *A. alternata* elicitation, MeJA treatment itself could not induce scopoletin production, indicating that another JA-independent signal is also needed for *NaF6ʹH1* activation.

Taken all together, the data strongly suggests that when infected with *A. alternata*, *N. attenuata* plants increase JA production, activate JA signalling through COI1, and then stimulate scopoletin biosynthesis for defence possibly by binding of MYC2 and some unknown transcriptional factors to the promoter region of *NaF6ʹH1* (Fig. 8). However, without *A. alternata* elicitation, MeJA treatment itself could not induce scopoletin production (Supplementary Fig. S5 at *JXB* online), indicating that another signal produced by fungal challenge is also needed for the induction. More efforts are required to understand the complex system for the regulation of scopoletin biosynthesis.

## Supplementary data

Figure S1. Alignment of NaF6ʹH1 and AtF6ʹH1.

Figure S2. Induction of scopolin in VIGS NaF6ʹH1 plants and of scopoletin in irCOI1 and irAOC plants.

Figure S3. Effect of ABA signalling on scopoletin accumulation.

Figure S4. T/G box in the *NaF6ʹH1* promoter.

Figure S5. MeJA alone cannot induce scopoletin.

Table S1. Gene-specific primers used for real-time PCR.

Supplementary Data
