# Supplementary material for: Scopoletin is a phytoalexin against Alternaria alternata in wild tobacco dependent on jasmonate signalling
Source: J Exp Bot. 2014 May 12;65(15):4305–15. doi: 10.1093/jxb/eru203 (PMC4112635; doi:10.1093/jxb/eru203)
Supplement: Supplementary Data [file supp_65_15_4305__index.html]

Scopoletin is a phytoalexin against Alternaria alternata in wild tobacco dependent on jasmonate signalling — Scopoletin is a phytoalexin against Alternaria alternata in wild tobacco dependent on jasmonate signalling — Supplementary Data 

# Scopoletin is a phytoalexin against *Alternaria alternata* in wild tobacco dependent on jasmonate signalling

## Supplementary Data

Data files

**Files in this Data Supplement:**

- Supplementary Data - Supplementary Data
