# Supplementary material for: Scopoletin is a phytoalexin against Alternaria alternata in wild tobacco dependent on jasmonate signalling
Source: J Exp Bot. 2014 May 12;65(15):4305–15. doi: 10.1093/jxb/eru203 (PMC4112635; doi:10.1093/jxb/eru203)
Supplement: Supplementary Data [file supp_eru203_jexbot122804_file001.pdf]

Scopoletin is a phytoalexin against *Alternaria alternata* in wild tobacco dependent on JA signaling

Huanhuan Sun[1], Lei Wang[1], Baoqin Zhang[2], Junhong Ma[3], Christian Hettenhausen[1], Guoyan Cao[1], Guiling Sun[1], Jianqiang Wu[1] and Jinsong Wu[1*]

[1]Key Laboratory of Economic Plants and Biotechnology, Kunming Institute of Botany, Chinese Academy of Science, Lanhei Road 132, 650201, Kunming, China

[2]Dalian Institute of Chemical Physics, Chinese Academy of Sciences, 457 Zhongshan Road, Dalian 116023, China

[3]Yunnan Academy of Tobacco Agricultural Science, Yuantong Street 33, 650031, Kunming, China

***Corresponding author:**

Jinsong Wu

Key Laboratory of Economic Plants and Biotechnology,

Kunming Institute of Botany, Chinese Academy of Sciences,

Lanhei Road 132, 650201 Kunming，P.R. China

Phone: 86-(0)871-65229552

Fax: 86-(0)871-65238769

Email: jinsongwu@mail.kib.ac.cn

Supplementary data

Fig. S1

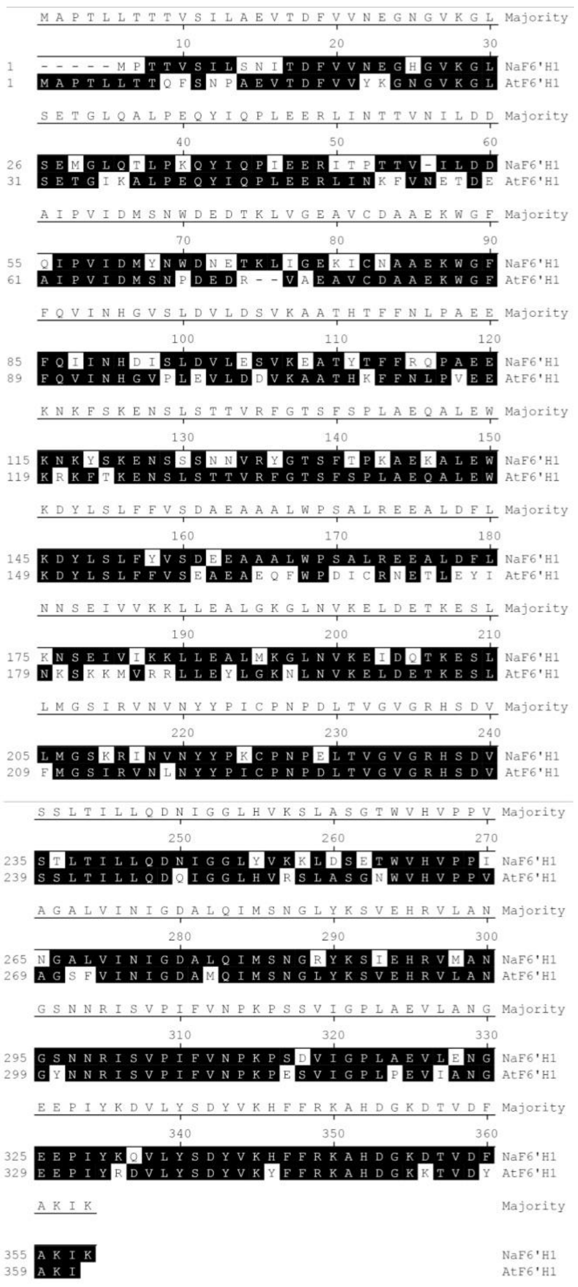

Fig. S1 Alignment of NaF6’H1 and AtF6’H1

The amino acid sequence of NaF6’H1 (KF771989) compared with AtF6’H1 (At3g13610); identical sequences are shaded with black.

Fig. S2

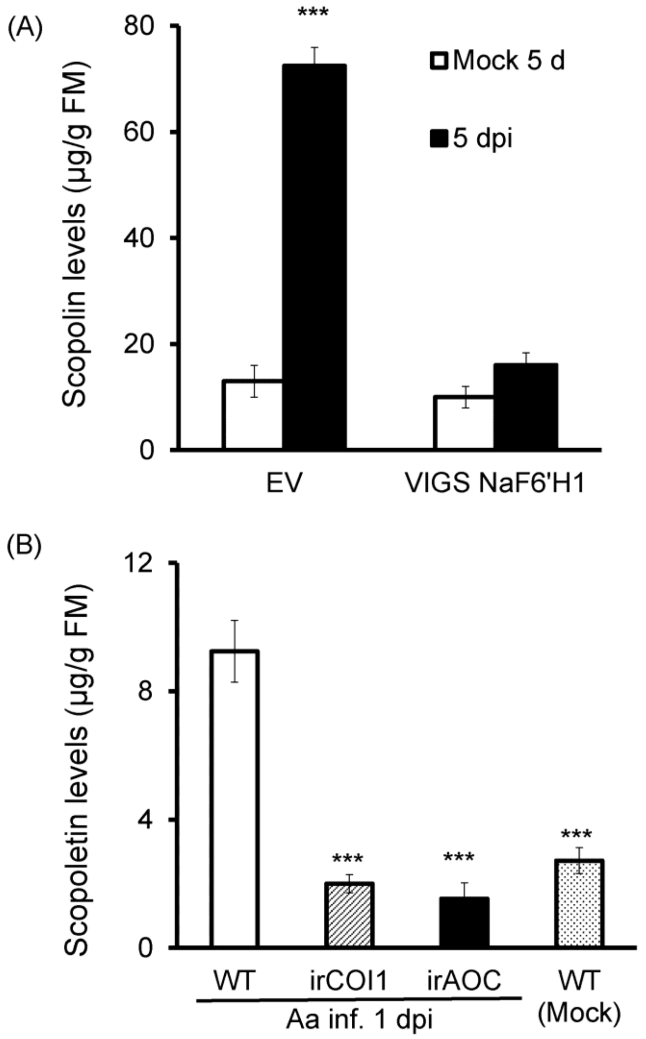

Fig. S2 Induction of scopolin in VIGS NaF6'H1 plants, and scopoletin in WT, irCOI1 and irAOC plants

(A): Mean (± SE) scopolin levels were determined by LC-MS/MS in 5 replicated leaves of EV and VIGS NaF6'H1 infected with *A. alternata* for 5 d.

(B): Mean (± SE) scopoletin levels were determined by LC-MS/MS in 5 replicated 0 leaves of WT, irCOI1 and irAOC plants at 1 dpi, and WT mock controls.

Asterisks indicate the level of significant differences between mock and infected samples in EV plants, and between WT and irCOI1 or irAOC plants at 1 dpi

(Student’s *t*-test: * $p<0.05$; *** $p<0.0001$).

Fig. S3

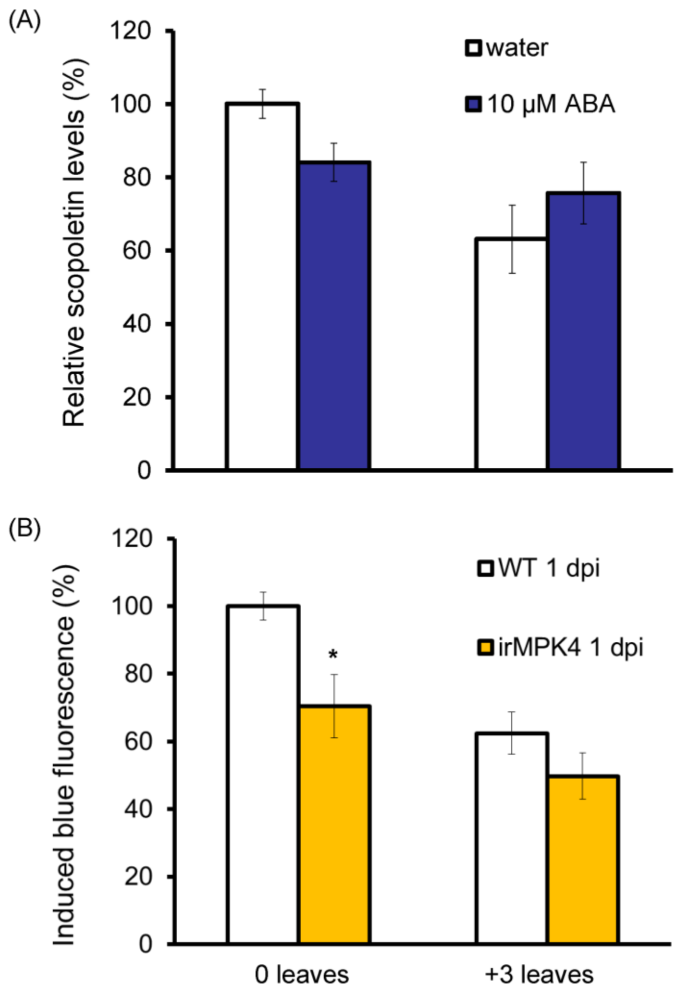

Fig. S3 Effect of ABA signaling on scopoletin accumulation

(A): Relative blue fluorescence intensity induced by *A. alternata* at 1 dpi in 4-replicated 0 and 3 leaves, which were pretreated with water or 10 µM ABA through petiole-feeding for 4 h. The level of the intensity in 0 leaves of WT pre-treated with water was arbitrary set as 100%.

(B): Relative blue fluorescence intensity induced by *A. alternata* at 1 dpi in both 4-replicated 0 and 3 leaves of WT and irMPK4 plants. The level of the intensity in 0 leaves of WT was arbitrary set as 100%.

Fig. S4

NaF6'H1 promoter has one T/G-box

GAAAATTTCCCTTGAAAGTGAGAGAGAGATAGGAGCTGGCAGCCAAGA
AAAACAAAATAAGGTTAGCTGGAATGGAAATCATAGGAAACAAACTTTGT
GTTTTTGTGTCCGTACATGCCGGCGGCCACAGGTTATAGGAAAACAGAC
TTACTAGGACTTTTGGCGAAGACTCTCAAACTTGTTTCAACACGTTTTGA
TAACCTCGTACACCTGGTTCCTAATTTTCTTCTGCTATATTTTAATTTCTTT
TTGTGAAAGATCGTCTTTTTTATTACTTCTGCTTTGGTCTCTTTAGGTCAA
CCAACTATTCATATATTAATTTGAACTTAATATTTGTATTGAAATAAGAACCA
CAATAGCCTAGACAAGGAGATCTACATCAGGATACATGCTAATATCTGAG
TTAGAACAAAAAAAAAGAGGAAAAGCTCTAGATATAAAAATAATTTTATTTT
TTTGGATTATTGAAAGCTAAATGTTTGTTTCTTCATTTCATTTTTATAGAAA
ATAGGATCTTTATTTTCTTATGGTTATAATTAGAATCAAAGGCGAGGTCGA
TAGTTGTTTCACTGTCGTTGCTATACGAATAAAAGACGTATATATGGTCAC
AGAAATTTAACATTTCCCACCATCCCTTCTAAGAGAAAAATAGACATGGA
AAATATTTTGTAATTTATTTTCACAATGAAGAGACAATTCCTGCACTAAATT
AGACTTGGTTTAGGCGTAAAAAGATACTAGACACAATTTTAATGTAATTTT
CTTAAATTTTTGTAACCTAATATAATTCTGAATTTTACCTTAAAATTCAACC
CTAAAACAAAGGTTGAAACTTCAAACGAATTAAATCA**AACGTGT**TCCTCG
TTTACTCAAAGGGACAAGTACCTATTGAACAAAGAAAAGTAAACGTAACA
TATTAACCCTATGAATACACAACAAGAAAATGTGCATGCACCAATCACATC
ATTTATTTTTCCACTGAACTAAAAATTCAGAACGTTAATTATTGAGAGTTTC
ATGGCATGACAATTGCCTATAAATACTGCACTAACTCAGAATCAATAGATC
ATCAAAGTTTTCCTCATTCAATTCATTTTTCATAAACCCTAATTTTCTCTTA
TTATTTCTTTTACTAATACAAAA**ATG**

T/G box: AACGTGT
Starting code: ATG

Fig. S4 T/G box in *NaF6'H1* promoter

NaF6'H1 promoter region was cloned and sequenced, potential MYC2 binding site of T/G box are indicated in red.

Fig. S5

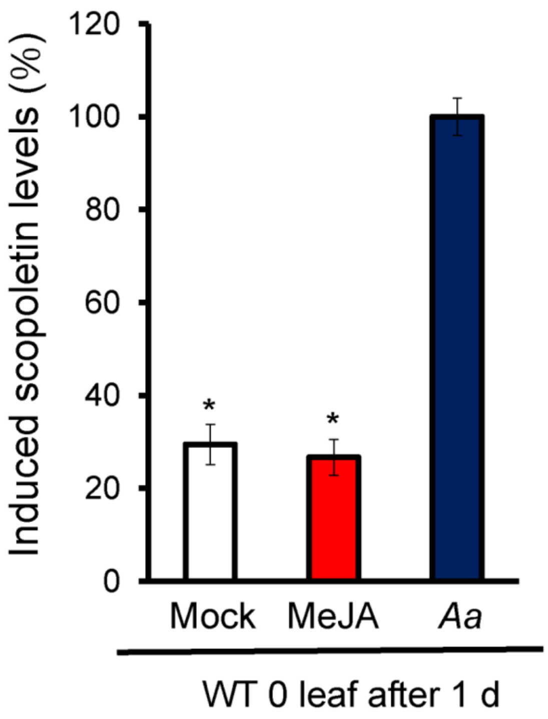

Fig. S5 MeJA alone cannot induce scopoletin

Mean (± SE) relative scopoletin levels were determined by LC-MS/MS in 5 replicated 0 leaves of mock, MeJA-treated and *A. alternata*-infected leaves at 1 dpi. The level of 0 leaves at 1 dpi was arbitrary set as 100%. Asterisks indicate the level of significant differences between *A. alternata* infected samples and mock or MeJA treated samples.

Supplementary table: Gene-specific primers used for real time PCR of *NaF6'H1* and *NaMYC2* genes.

NaActin_F	5'- GGTCGTACCACCGGTATTGTG -3'	(Wu et al. 2013)
NaActin_R	5' –GTCAAGACGGAGAATGGCATG -3'	(Wu et al. 2013)
NaF6'H1_F	5' –CGAGTTATGGCTAATGGCAGC-3'	This study
NaF6'H1_R	5'- AGCACTTCAGCCAAAGGACC-3'	This study
MYC2_rF	5'- GAGGTGGCGATTCGGATCAA -3'	(Woldemariam *et al.*, 2013)
MYC2_rR	5'- CTCGGCTTCTTCTCAGGGTC -3'	(Woldemariam *et al.*, 2013)
